# Trichinellosis Caused by Consumption of Wild Boar Meat — Illinois, 2013

**Published:** 2014-05-23

**Authors:** Yoran Grant Greene, Thomas Padovani, Jo Ann Rudroff, Rebecca Hall, Connie Austin, Michael Vernon

**Affiliations:** 1 EIS officer, CDC; 2 Illinois Department of Public Health; 3 Cook County Department of Public Health, Chicago, Illinois; 4 Missouri Department of Health and Senior Services; 5 Division of Parasitic and Malarial Diseases, Center for Global Health, CDC

On March 6, 2013, the Cook County Department of Public Health (Chicago, Illinois) contacted the Illinois Department of Public Health regarding a diagnosis of trichinellosis in a patient who had consumed wild boar and deer meat obtained by hunting at a Missouri ranch January 16–18. Trichinellosis is a parasitic infection caused by consumption of undercooked infected meat, most commonly from carnivorous or omnivorous animals (1).

The Cook County and Illinois health departments and the Missouri Department of Health and Senior Services queried the Illinois and Missouri electronic reportable disease registries and interviewed patients to identify additional cases and describe patients’ clinical characteristics. CDC performed immunoglobulin G enzyme-linked immunosorbent assay testing of patient serum and microscopically examined the meat for evidence of *Trichinella* larvae.

Patient interviews revealed that the index patient had ground the wild boar and deer meat into sausage and served it to three family members who had participated in the hunt. The sausage was shared with a friend and the friend’s four family members, none of whom had participated in the hunt. A case was defined as illness in a person who consumed the implicated meat and had positive serology or myalgias. Nine cases were identified. All nine persons had consumed the implicated sausage during January 20–February 16 and experienced illness compatible with trichinellosis during February 13–March 4; three of six tested had a positive serologic test for antibodies specific to *Trichinella* within 7 days of symptom onset. No one else consumed the sausage, and no additional cases were identified from electronic disease registries.

Among the nine cases, five occurred among men (median age = 35 years; range = 20–54 years), and the median incubation period was 16 days (range = 4–24 days). All patients reported myalgias, eight had periorbital edema, and seven had both fever and eosinophilia. *Trichinella spiralis* larvae were identified microscopically in the sausage but not in the deer meat, indicating that the boar meat was the likely source. All patients were treated solely with albendazole and recovered without complications.

Trichinellosis cases remain infrequent in the United States because of state and federal laws preventing feeding of uncooked swill to commercial swine and public awareness of the danger of eating raw or undercooked game meat. The Missouri Department of Health and Senior Services provided additional education to employees of the ranch about the risk for *Trichinella* ingestion and the need to inform hunting patrons. The Illinois Department of Public Health recommends posting advisories at hunting ranches that inform hunters of the importance of cooking game meat to the cooking temperature of 71°C (160°F) recommended by the U.S. Department of Agriculture and CDC before consuming it (2).
